# Granuloma after breast conserving surgery—a report of three cases

**DOI:** 10.1093/jscr/rjab199

**Published:** 2021-06-04

**Authors:** Yuki Ichinose, Yoshimasa Kosaka, Toshiaki Saeki, Akihiro Fujimoto, Asami Nukui, Aya Asano, Hiroko Shimada, Kazuo Matsuura, Takahiro Hasebe, Akihiko Osaki

**Affiliations:** Department of Breast Oncology, Saitama Medical University International Medical Center, Saitama, Japan; Department of Breast Oncology, Saitama Medical University International Medical Center, Saitama, Japan; Department of Breast Oncology, Saitama Medical University International Medical Center, Saitama, Japan; Department of Breast Oncology, Saitama Medical University International Medical Center, Saitama, Japan; Department of Breast Oncology, Saitama Medical University International Medical Center, Saitama, Japan; Department of Breast Oncology, Saitama Medical University International Medical Center, Saitama, Japan; Department of Breast Oncology, Saitama Medical University Hospital, Saitama, Japan; Department of Breast Oncology, Saitama Medical University International Medical Center, Saitama, Japan; Department of Breast Oncology, Saitama Medical University International Medical Center, Saitama, Japan; Department of Breast Oncology, Saitama Medical University International Medical Center, Saitama, Japan; Department of Breast Oncology, Saitama Medical University International Medical Center, Saitama, Japan

**Keywords:** Breast cancer, Granuloma, Breast conserving surgery, Ipsilateral recurrence

## Abstract

Granulomatous mastitis is a rare breast disease that is categorized as a benign tumor with chronic inflammation. Since the cause of the chronic inflammation is usually unknown, it is sometimes called idiopathic granulomatous mastitis (IGM). Although imaging modalities, such as ultrasound, magnetic resonance imaging and mammography can detect tumors, they are sometimes unable to differentiate between benign and malignant tumors. In such cases, biopsy is needed to make a correct diagnosis. We experienced three cases of IGM after breast conserving surgery in breast cancer patients in whom we needed to rule out recurrence of breast cancer. In our cases, tumorectomy was performed in two cases for pathological diagnosis, since neither biopsy nor cytology was able to reveal a conclusive pathological diagnosis. Our management of these three cases might suggest the appropriate management of granulomatous tumors after breast conserving surgery in breast cancer survivors.

## INTRODUCTION

Granulomatous mastitis is a rare breast disease that a benign tumor with chronic inflammation [1]. Since the cause of the chronic inflammation is usually unknown, it is sometimes called idiopathic granulomatous mastitis (IGM). Although imaging modalities, such as ultrasound, magnetic resonance imaging (MRI) and mammography can detect tumors, they are sometimes unable to differentiate between benign and malignant tumors [2]. Here, we report three cases of IGM after breast conserving surgery (BCS) in breast cancer patients.

## CASE REPORT

Case 1: The patient was a 69-year-old woman who was diagnosed with right breast cancer. She underwent right partial mastectomy with sentinel lymph node biopsy in 2010. Pathological finding was a non-invasive ductal carcinoma, that was margin negative, estrogen receptor positive, progesterone receptor positive and human epidermal growth factor receptor 2 (HER2) negative. She subsequently underwent 50 Gy irradiation of the ipsilateral breast, followed by tamoxifen for 5 years.

**Figure 1:**
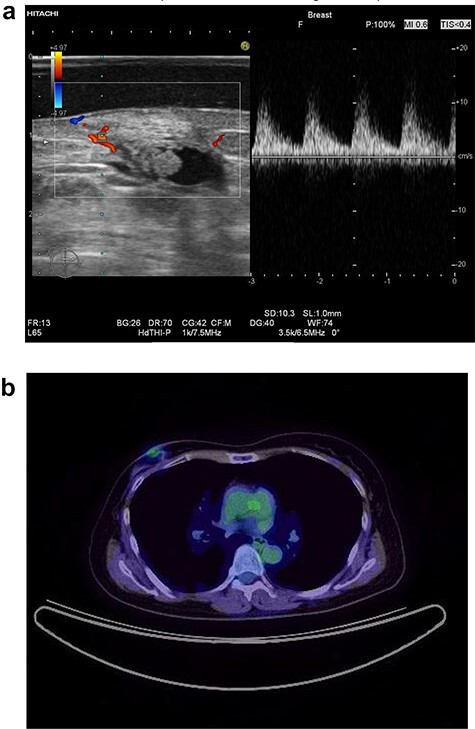
Ultrasound of the right breast in case 1 revealed a mass-like lesion 16.6 × 10.0 × 4.9 mm (**a**), and PET-CT showed that the right breast mass had an SUV max of 2.7 (**b**).

In 2014, a right breast tumor was located at the site of the surgical scar with slight erythema. Ultrasound revealed a hypoechoic mass with an irregular shape, indicating possible recurrence of breast cancer (Fig. 1a). Positron emission tomography–computed tomography (PET-CT) also suggested a right breast tumor with a relatively high standardized uptake value (SUV) and no distant metastasis (Fig. 1b). She underwent tumorectomy for both tumor removal and to obtain a pathological diagnosis, since she was afraid of false negative results with core needle biopsy (CNB). Pathological finding was a xanthogranulomatous lesion with cystic changes and dense sclerosis without any evidence of malignancy. There was no recurrence after surgery.

Case 2: A 61-year-old woman who was diagnosed with left breast cancer underwent left partial mastectomy with sentinel lymph node biopsy in 2013. Pathological finding was an invasive ductal carcinoma (12 mm, pT1c) that was margin negative, pN0, estrogen receptor positive, progesterone receptor positive, and HER2 positive. She received EC (epirubicin and cyclophosphamide) followed by docetaxel and trastuzumab then 50 Gy irradiation of the ipsilateral breast followed by letrozole for 5 years.

**Figure 2:**
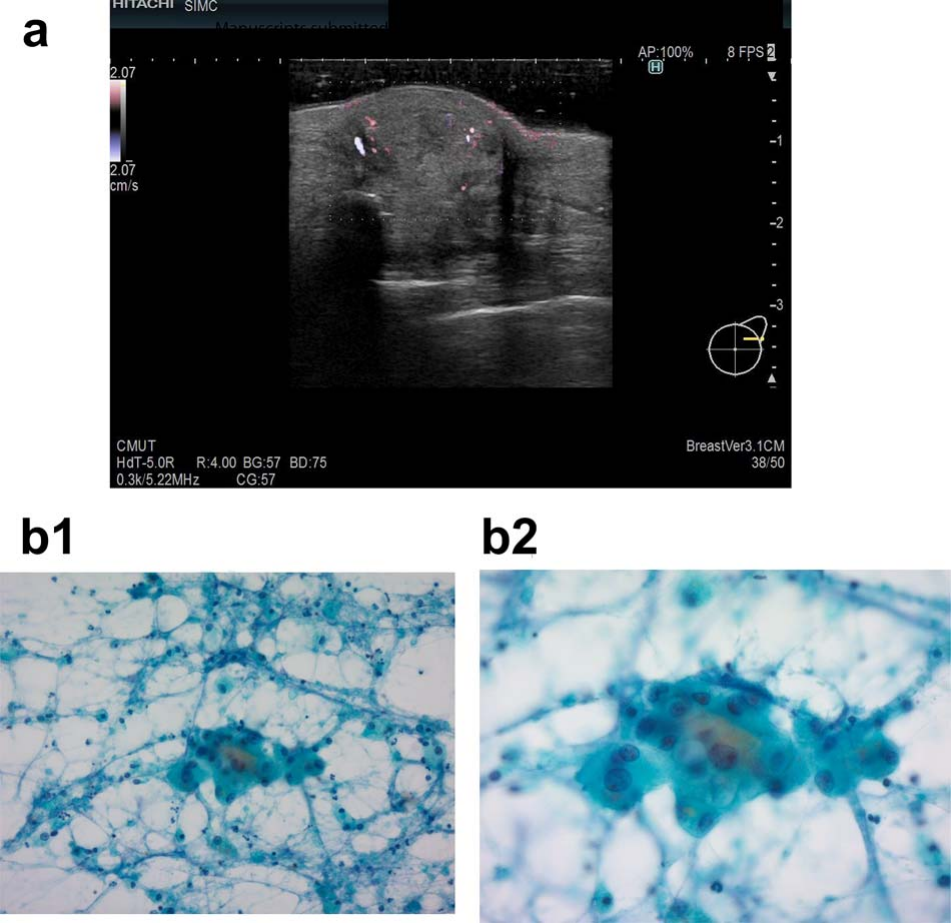
Ultrasound examination in case 2 revealed a mass 19.4 × 18.8 × 10.7 mm in the left breast (**a**) and cytodiagnosis did not unclear that it showed local recurrence. (**b-1**: ×20 magnification, **b-2**: ×40 magnification).

In 2019, she found a left breast tumor located at the site of the surgical scar, with marked erythematous changes of the skin. Ultrasound revealed a hypoechoic cystic mass with an irregular shape, indicating possible recurrence of breast cancer (Fig. 2a). Cytology was suspicious for carcinoma recurrence (Fig. 2b). PET-CT detected a left breast tumor and no distant metastasis. She underwent tumorectomy for both removal of the tumor and pathological diagnosis.

Pathological finding was a xanthogranulomatous inflammation and calcification without any evidence of malignancy. There was no recurrence after surgery.

Case 3: A 60-year-old woman who was diagnosed with right breast cancer underwent right partial mastectomy with axillary lymph node dissection in 2008. Pathological finding was an invasive ductal carcinoma (15 mm, pT1c), that was margin positive (<2 mm), pN2 (6/15), estrogen receptor positive, progesterone receptor positive and HER2 negative. She received EC followed by docetaxel, and 54 Gy irradiation with 10 Gy boost. Subsequently, she had taken tamoxifen for 4 years and exemestane for 6 years.

**Figure 3:**
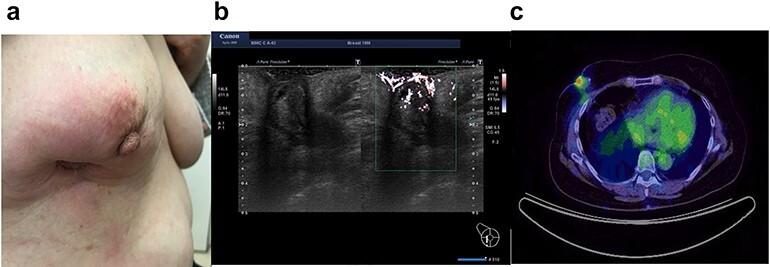
Case 3 presented with erythematous changes in the skin of the right breast (**a**). Ultrasound evaluation revealed a mixed pattern mass lesion 26.3 × 34.8 × 25.0 mm in the right breast (**b**), and PET-CT showed a right breast mass with an SUV max of 4.0 (**c**).

**Figure 4:**
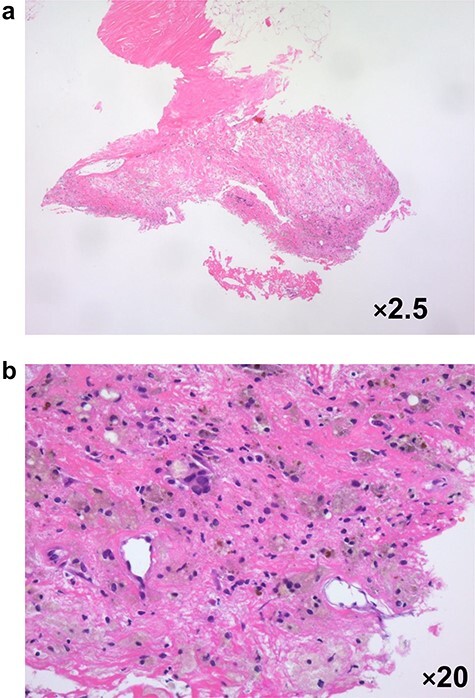
Pathological evaluation of a vacuum-assisted core needle biopsy specimen in case 3 revealed xanthogranulomatous inflammation with micro-calcification **a**: ×2.5 magnification, **b**: ×20 magnification.

In 2020, she complained of right breast pain. She also noticed erythematous changes of the skin on her right breast. Examination revealed a right breast tumor at the site of the surgical scar with markedly erythematous changes of the skin (Fig. 3a). Ultrasound revealed a hypoechoic cystic mass, resembling a chronic abscess with thick skin. The cystic nature of the tumor suggested the possibility of recurrence of breast cancer (Fig. 3b). PET-CT revealed a right breast tumor and no distant metastasis, which was suspicious of local recurrence (Fig. 3c). Laboratory investigation indicated a C-reactive protein level of 2.3 mg/L and white blood cell count of 8770/mL. She was administered antibiotic therapy, consisting of cefaclor for 7 days, which resulted in the disappearance of the erythema. Subsequently, she underwent vacuum-assisted core needle biopsy (VACNB) of the wall of the cystic tumor. Pathology finding was a xanthogranulomatous inflammation and necrotic tissue with scar formation without any evidence of malignancy (Fig. 4). Additional VACNB was performed, which showed no malignancy. She elected not to undergo excision biopsy. Follow-up ultrasonography performed after 1.5 months, VACNB revealed no abnormal findings.

## DISCUSSION

IGM can clinically resemble an abscess or breast cancer [1]. Although IGM is benign, it can cause ulceration and thickening of the breast skin. Since the optimal treatment of this condition has not been established [3], surgeons usually opt for surgery in these cases to rule out recurrence of malignancy [4]. The incidence of breast biopsy following treatment for breast cancer is not well characterized. The two patients opted for surgical excision of the tumor. The third patient decided not to undergo excision biopsy, because she did not want additional surgery. In a study of 2065 patients, Law *et al*. [5] demonstrated that one in five patients required breast biopsy during post-treatment surveillance following BCS, most of which were benign tumors. Zhao *et al*. [2] reported that whole-lesion histogram and texture analysis using apparent diffusion coefficient provides a non-invasive analytical approach to differentiate between IGM and invasive breast cancer, both of which present with non-mass enhancement without rim-enhanced masses. Additionally, in cases of local recurrence, the rates of subsequent mastectomy due to ipsilateral or contralateral malignancy are low [6]. Davis *et al*. [7] reported that IGM is a self-limited, benign condition that requires no treatment, and that just observation without resection should be enough. After diagnosis, surgical treatment can be limited to drainage procedures for fluid collections. In our cases, we needed to consider sampling errors in VACNB and our patients’ preferences in determining the appropriate method to rule out recurrence.

While granulomas should be considered in the differential diagnosis of lumps at the site of the previous surgical scar in breast cancer survivors, local recurrence in the conserved breast needs to be ruled out. VACNB might be useful in such cases. If the tumor is benign, follow-up observation without any further treatment might be reasonable. However, in some cases, tumorectomy should be allowed depending on the patient’s preference.
